# An evaluation of the Nova 2 ionised calcium instrument

**DOI:** 10.1155/S1463924680000296

**Published:** 1980

**Authors:** J. A. Fyffe, A. S. Jenkins, H. N. Cohen, F. J. Dryburgh, M. D. Gardner

**Affiliations:** 1 University Department of Medicine Royal Infirmary Glasgow G4 0SF Scotland; 2 Department of Biochemistry Royal Infirmary Glasgow G4 0SF Scotland


Many enzyme assays are linked to the formation or utilisation of NADH so that reaction rates can be monitored at 340 nm. The absorbance spectrum of naphthol green is sufficiently different from that of NADH to render calibration using this substance undesirable, and NADH or the particular chromophore used to monitor the reaction, e.g. p-nitrophenol, should be used.

The new calibration procedure confirmed the extent of the disparity between the two instruments noted when performing enzyme analyses and supported the view that the problem was one of instrumentation, possibly electronic in origin. An electronic calibration procedure produced by the company was successful in bringing the instruments back into specification and cured the problem of the disparity between the 0.05 and 0.20 absorbance scales.

Exactly what is accomplished when electronic calibration is carried out remains uncertain. Certainly such items as the ratio of the two absorbance scales and linearity across the recorder are correctly adjusted during this procedure, but the manner in which the photocell output is linked to true absorbance is not clear. In the light of the authors' experience, it is difficult to avoid the conclusion that electronic calibration merely succeeded in ensuring that both instruments investigated produced the same result. It may not ensure that the output from an instrument is linked to true absorbance.

For this reason, a correction factor may still need to be applied to the results obtained after electronic and absorptiometric calibration has been carried out. It is suggested that results which are within ± 5% of the true absorbance need not be corrected, while instruments operating outside of these limits should have a correction factor applied until they can be serviced and the error corrected. This may well depend on the type of analysis being performed on the instrument.

The calibration procedure outlined could be performed at intervals of about six months. It can be adapted to calibrate any other type of reaction rate analyser and has been used successfully in calibrating both the AKES (MSE Scientific Instruments, Manor Royal, Crawley, West Sussex), and Centrifichem (Union Carbide (U.K.) Ltd., Meteor House, White Lion Road, Amersham, Bucks) systems. If such a proposal were generally adopted, better inter-laboratory agreement of enzyme results should occur. For future instruments, recommendations have been made which set very high specifications [5] and which should render future calibration a more precise and rapid exercise.

ACKNOWLEDGMENT
The authors would like to thank Dr. H. G. Sammons for his interest and advice.

REFERENCES

[1] Instruction manual to Reaction Rate Analyser 2086 Appendix VIII LKB Produkter A.B. Sweden.
[2] Tietz, N. W. (Ed.), 1976, Fundamentals of Clinical Chemistry, 2nd Edition, W. B. Saunders & Co. Philadelphia.
[3] Moss, D. W., 1976, News Sheet No. 159, Association of Clinical Biochemists.
[4] Burke, R. W., Deardorff, E. R. and Menis, O. 1972, Liquid Absorbance Standards, *J. Research. Nat. Bureau of Standards A. Physics and Chemistry*, 76A, 469.
[5] Instrumentation Guidelines Study Group, 1977, Guidelines for photometric instruments for measuring enzyme reaction rates, *Clinical Chemistry*, 23, 2160-2162.

### An evaluation of the Nova 2 ionised calcium instrument

**J.A. Fyffe, A.S. Jenkins and H.N. Cohen**
*University Department of Medicine, Royal Infirmary, Glasgow G4 OSF*

**F.J. Dryburgh and M.D. Gardner**
*Department of Biochemistry, Royal Infirmary, Glasgow G4 OSF*

### Introduction

The hypothesis of McLean and Hastings [1] that free or ionised calcium is the physiologically active fraction of plasma calcium is now well accepted. Various methods of measuring this have been used including bioassay [1,2] bioluminescence [3,4] ultra-filtration [5] and ion-selective electrodes [6-13] Ion-selective electrodes have been available for nearly twenty years and specific versions for use in the clinical laboratory for more than ten years. Many of these however have serious shortcomings. They are difficult to set up and have a short membrane life. Once set up they provide a simple and rapid measurement of ionised calcium.

The Nova 2 instrument shown in Figure 1 is manufactured by Novabiomedical, Newton, Mass. U.S.A. and marketed in the United Kingdom by American Hospital Supply (U.K.) Ltd., Didcot, Oxon. The electrode assembly consists of a calcium selective electrode and a silver-silver chloride reference electrode with a KCl bridge. The calcium selective electrode is housed in a plastic box containing internal filling solution (calcium chloride) in a gel form. The standard and test solutions flow through the teflon tube which passes through the gel.

In the teflon tube is an ion-selective window which acts as a membrane. The inner surface of the window is coated with a calcium polyphosphate ion exchanger in a non-aqueous medium. An internal silver/silver chloride electrode connects the ion-selective electrode by a silver wire to the electronic circuit.

The silver/silver chloride reference electrode consists of a silver wire embedded in a silver chloride pellet. The internal reference solution (2 M KCl) flows past this to meet the sample stream in a dynamic liquid junction. The reference electrode also incorporates a pair of platinum electrodes which sense the presence of air or liquid and are used by the computer to monitor cycle performance. The electrodes are mounted by a simple plug-in device in a heated aluminium block which is maintained at a temperature of 37°C. A calcium electrode, a reference electrode and a spare calcium electrode (these are guaranteed for six months use provided Nova fluids packs are used) are supplied with the instrument.

The functions of the analyser are controlled by a small in-built computer and selection of the 'calibrate' or 'analyse' cycle is by simple push buttons. The instrument has two operating modes, 'stat' and 'stand by'. In the 'stat' mode the instrument automatically calibrates every two hours and is stated to be ready for immediate use. 'Stand by' maintains temperature and slow circulation of fluids in the analytical section. In this mode, which is the more economical of reagents, it is necessary to calibrate before use but this takes only a few minutes.

In the calibration cycle, the computer controls and monitors the flow of standards A and B (1.0 and 2.0 mM $CaCl_2$ respectively) and reference solution (2M KCl) to the electrodes. To maintain an ionic strength equivalent to that of serum, the standards are dissolved in 150 mM NaCl. Using the millivolt readings of the two standards taken at a fixed time, the computer calculates a calibration line. This is compared with in-built criteria based on an unmodified Nernst equation and, if unsatisfactory, causes an error message to appear. If the slope and stability of readings are acceptable, the 'ready' light comes on and the 'analyse' cycle can be initiated. The first push of the 'analyse' button causes the motor-driven probe to be presented and a second push aspirates the sample. The instrument then aspirates standard A and uses the reading to alter the position (but not the slope) of the calibration line, if necessary, before calculating and presenting the result of the sample. This aspiration of standard A also washes the sample through along with air segments.

The probe has two sampling positions: in the first it aspirates from a syringe, capillary tube or open container and in the second will pierce the stopper of a vacuum tube and, having vented the tube, will aspirate the sample. When retracted, standards A or B can be sampled as shown in Figure 2.

The flow of standard and reference solutions (provided in a Fluids Pack)* and air for segmentation is controlled and monitored by the computer via a variable speed peristaltic pump with planetary gears and a fluidic valve.

The sampler, electrodes and pump are connected by plastic tubing and the flow-through system, Figure 2, is designed so that the narrowest bore is in the probe. If a blockage occurs from fibrin threads etc. it is easily removed from the probe. It is recommended that the tubing is changed every six months and a complete system is available from the manufacturers.

Results are displayed as mg/100 ml or mmol/l. As an alternative, the displays can show mV, heating block temperature, electrode slope and instrument status code. The status codes of the instrument are shown in Table 1.

**Table 1 Nova 2 instrument status codes**

| Status Codes | Indication |
|---|---|
| 2 | Analysis in progress |
| 4 | Calibration in progress |
| 5 | Purge in progress |
| 6 | Automatic calibration in progress |
| 7 | Automatic calibration (repeat) in progress |
| 8 | System idle, no errors |
| 21 | Analog-to-digital converter overload |
| 22 | Sampler out of position |
| 26 | Signal drift |
| 28 | Electrode slope out of range |
| 40 | Sample holder position error |
| 41 | Sampler probe position error |
| 42 | Valve position error |
| 49 | Math error |
| 56 | Instability of calcium reading |
| 58 | Air when not allowed |
| 59 | No air when required |
| 61 | Not calibrated |
| 62 | Temperature low |
| 63 | Temperature high |
| 64 | Stand by mode |
| 70 | Converter error |
| 77 | Program error |

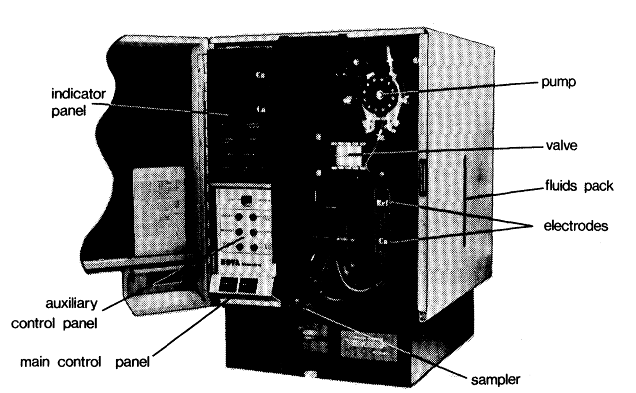

*Figure 1 The Nova 2.*

### Instruction manual

The 80 page instruction manual includes sections on the principles of ion-selective measurement, setting up and use of the instrument, explanations of the controls, flow diagrams and a useful section on trouble-shooting.

### Warranty and servicing

The manufacturers warranty covers the instrument for one year and the electrodes and tubing harness for six months. After the warranty period, a service contract is available at approximately 8% of the instrument cost.

### Evaluation

As far as was practicable the recommendations of the International Union of Pure and Applied Chemistry were followed [14]. While the instrument was being evaluated a second Nova 2, with two calcium electrodes, was available on loan from the suppliers. The performance of each instrument was compared.

**The Fluids Pack comprises 500 ml each of standards A and B and reference solution and a waste container with disinfectant. Solutions are supplied only as a complete pack and are sufficient for approximately 500 tests depending on the frequency of calibration.*

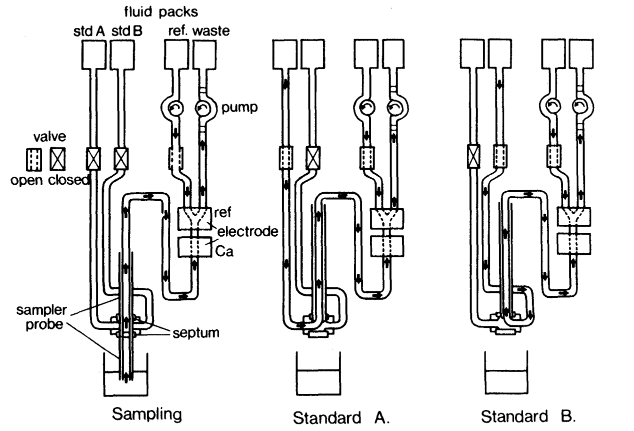

*Figure 2 Flow diagrams.*

### Results

### Aqueous solutions

No significant difference was found in the response of any of the four calcium electrodes to pure solutions when used in either instrument.

*Calibration curve* A typical curve is shown in Figure 3 and is linear from 0.5 to 5.0 mM Ca. This covers the expected physiological range adequately. The calibration curve was prepared using solutions of $CaCl_2$ in 150 mM NaCl.

*Limit of detection* This was less than 0.05 mM for the electrodes examined.

*Practical response time and drift* These were measured by reading the mV display using a 1.0 mM Ca standard following an analyse cycle. The results are shown in Figure 4. The reading reaches 90% of its final value in approximately 33 seconds and the instrument takes the reading at 44.5 seconds. Stability is reached after 5 minutes and thereafter the reading is steady for at least a further 10 minutes.

*Interfering substances* Potential interfering substances were selected following the criterion described by Robertson [5]. These were zinc, strontium, magnesium, barium, manganese, sodium and potassium. The effect of hydrogen ion was not studied as its relative concentration in blood (nM) would be expected to cause minimal interference. Since the standards supplied with the instrument contain sodium (150 mM), the studies on the other ions were carried out on 150 mM NaCl solutions.

The electrodes responded to zinc (1.5 μM), and magnesium (1.2 mM) which gave apparent calcium results above the limit of detection for calcium. Responses for strontium (20 μM), barium (20 μM), manganese (20 μM), sodium (140 mM) and potassium (5mM) were below the detection limit for calcium. Further investigation of the effects of zinc and magnesium at approximately physiological levels were carried out using the fixed interference method where the e.m.f. of the cell is measured with solutions of constant level of interference (Zn 1.5 μM, Mg 0.6 and 1.2 mM) and varying concentration of calcium. The potentiometric selectivity co-efficient $\underline{K}^{Pot}_{A,B}$ was calculated for each interfering ion and results for a typical electrode are shown in Table 2. While sodium (140 mM) did not interfere above the detection limit for calcium and gave a very small $\underline{K}^{Pot}_{A,B}$ when used in the fixed interference method, the apparent calcium result was increased by 2–3% on reducing the sodium concentration from the 150 mM used in the standards to 120 mM. This may be an ionic strength effect since it is well known that this alters the liquid junction potential.

The instrument does not compensate for alterations in liquid junction potential and the design of the instrument does not allow this parameter to be investigated further.

**Table 2. Potentiometric selectivity coefficients for electrode 1**

| Interfering ion (conc$^n$) | Ca conc$^n$ | $\underline{K}^{Pot}_{A,B}$ |
|---|---|---|
| Zn (1.5 μM) | 0.11 mM | 73 |
| Mg (0.6 mM) | 0.09 mM | 0.16 |
| Mg (1.2 mM) | 0.08 mM | 0.06 |

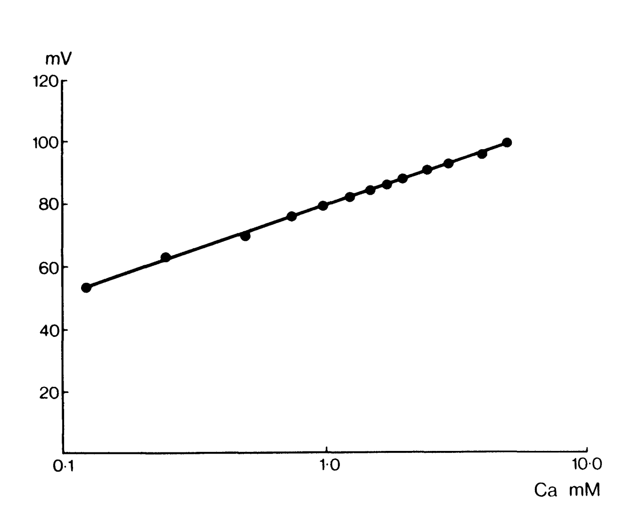

*Figure 3 A typical calibration curve obtained using solutions of $CaCl_2$ in 150 mM NaCl.*

*Precision* The within batch coefficient of variation (C.V.) of a 1.5 mM Ca standard was 0.5% and that between batch was 1.2%.

### Blood, plasma and serum

*Practical response time* This was measured by detaching the tubing from the probe and placing it in a serum sample so that serum was sampled at all stages of the analytical cycle. The mV reading was taken as described above for aqueous standards and results for two electrodes are plotted in Figure 5. Compared with aqueous standards the reading fell slowly and failed to reach a steady rate even after 45 minutes. It was not possible therefore to measure the practical response time for serum. In contrast to their responses to aqueous solutions, there were also marked differences between the two electrodes.

*Blood from laboratory staff* Preliminary studies were carried out on healthy laboratory staff in the age group 20–50 years. In the first study (using one instrument only) heparinised blood samples (14 units lithium heparin/ml) were taken without venous stasis and measured immediately. The samples were centrifuged and the plasma ionised calcium measured immediately. The results are shown in Table 3. No sex difference was observed. The precision of the instrument

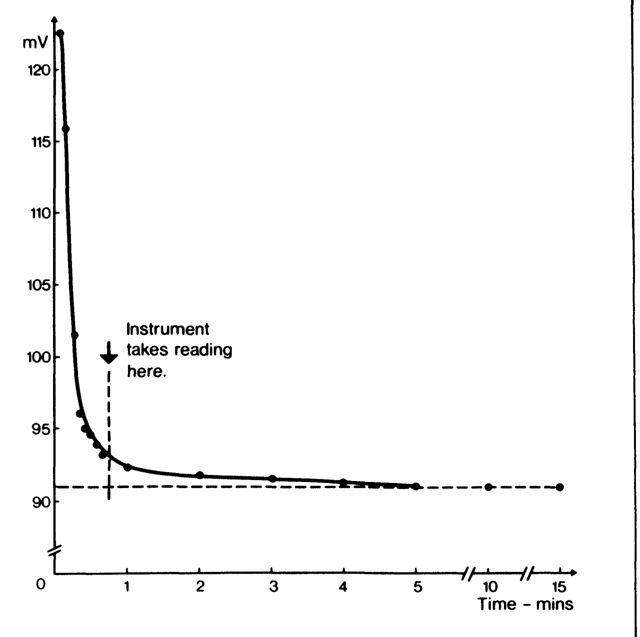

*Figure 4 The practical response time to aqueous standards.*

was established by dividing blood and plasma from a single subject into 15 small containers. While the specimens were not kept anaerobic, each was treated identically. Results are shown in Table 4.

During the next part of the study both instruments were used to establish provisional reference ranges. Blood was again taken from laboratory staff and analysed on each instrument. As well as heparinised blood and plasma, the ionised calcium of serum from blood which was allowed to clot for 30 minutes at room temperature was measured. Blood, plasma and serum from each subject was available and these were analysed in duplicate on each instrument with less than 1 minute delay between the measurements on each instrument. As no transport was involved, pH changes were minimised. The results (Table 5) show a significant difference between the instruments when measuring blood, plasma and serum and suggest that both instruments give results which vary with the type of specimen analysed. In order to study this further, replicate specimens of blood, plasma and serum from a single donor were measured on both instruments and the blood/plasma, blood/serum and plasma/serum results compared. All were significantly different ($p < 0.001$ in each case) as shown in Table 6. The between instrument difference was again evident and on exchanging calcium selective electrodes between instruments it was established that these differences were a function of the electrodes.

*Sample size*

This is quoted by the manufacturers as 350 $\mu$l, however it was found to be larger (approximately 400 $\mu$l) in these experiments.

*Subjective evaluation*

The instrument was easy to use and robust. During twelve months use no evidence was found of deterioration of the electrodes which appear to be unaffected by protein deposition. The electrodes are simple to change as they are merely plugged in, attached to the tubing harness and are ready to use as soon as they reach 37°C (about 10 minutes). While the fluid packs are expensive they contain only simple chemicals and could readily be prepared in the laboratory. The error code display, together with the table of status codes, obviates most spurious results which may arise (see Table 1). In the authors' experience, most problems can be overcome by recalibration. The instruction manual contains a large section on trouble-shooting but this was not needed in this study. Difficulties were encountered when using vacutainers in that standard solutions were occasionally sucked into the vacuum tube. This was due to a failure of the venting system which should fill the tube with air before the probe aspirates the sample.

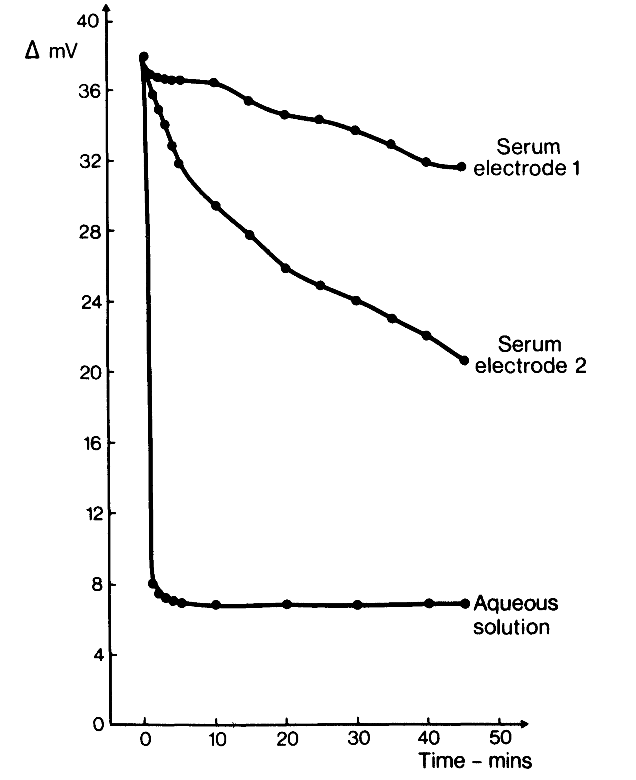

*Figure 5 Response time to serum (two electrodes) compared with aqueous solution.*

**Table 3. Results from healthy laboratory staff (Instrument 1)**

| | n | $\bar{x}$ | SD | Range (± 2 SD) |
|---|---|---|---|---|
| Blood | 24 | 1.163 | 0.074 | 1.02 – 1.30 |
| Plasma | 24 | 1.083 | 0.061 | 0.96 – 1.20 |

**Table 4. Precision of Instrument 1 – Replicate analyses on blood and plasma from a single subject.**

| | n | $\bar{x}$ | SD | CV |
|---|---|---|---|---|
| Blood | 15 | 1.226 | 0.036 | 2.9% |
| Plasma | 15 | 1.101 | 0.030 | 2.8% |

**Table 5. Between instrument differences on results from 20 subjects.**

| | Instrument 1 | | Instrument 2 | |
|---|---|---|---|---|
| | $\bar{x}$ | Range (± 2 SD) | $\bar{x}$ | Range (± 2 SD) |
| Blood* | 1.16 | 1.06 – 1.26 | 1.24 | 1.13 – 1.32 |
| Plasma** | 1.07 | 1.00 – 1.14 | 1.09 | 1.00 – 1.17 |
| Serum* | 1.16 | 1.11 – 1.21 | 1.21 | 1.15 – 1.27 |

Pair difference t test on each group showed that the between instrument differences of replicates were significant (*$p < 0.001$ and **$p < 0.1$).

**Table 6. Blood, plasma and serum from a single subject measured on two instruments**

| | Instrument 1 | | | | Instrument 2 | | | |
|---|---|---|---|---|---|---|---|---|
| | n | $\bar{x}$ | SD | CV | n | $\bar{x}$ | SD | CV |
| Blood | 20 | 1.181 | 0.021 | 1.8% | 19 | 1.253 | 0.045 | 3.6% |
| Plasma | 18 | 1.024 | 0.017 | 1.7% | 20 | 1.073 | 0.019 | 1.8% |
| Serum | 15 | 1.056 | 0.007 | 0.6% | 20 | 1.101 | 0.005 | 0.4% |

### Discussion

The authors were impressed by the speed and ease of use of the Nova 2. The electrodes appear robust and long lasting when compared with previous instruments [6-13]. The ion exchanger in the calcium electrode is affected by the presence of magnesium and zinc at physiological levels. It is suggested that these ions be included in the standards.

The practical response time is satisfactory for pure solutions but slow and variable for serum and this might account for the between electrode differences seen in Tables 4 and 5.

In the instruction manual the manufacturers quote the normal values found by Ladenson and Bowers [8] measured at 25°C on an Orion instrument and state that this range is for blood, plasma or serum thus taking no account of the heparin and erythrocyte effects, both of which are discussed by these authors in the paper from which the normal range is quoted. Values obtained in this study clearly show the blood, plasma and serum differences due to these effects. The depression of free calcium by heparin, using aqueous calcium solutions, was also confirmed. The precision was less than the quoted 0.5% on blood and plasma and best on serum. To overcome these factors, specimen collection was standardised. Vacuum tubes were used without anticoagulant. The blood was allowed to clot at room temperature and the serum sampled within an hour.

A disquieting feature is the difference between electrodes. If this degree of change in measured values were to occur each time an electrode was replaced it would be necessary to establish a reference range or a "correction factor" for each electrode. This was discussed with the manufacturers who have indicated that results on electrode response time are unacceptable and have offered to replace the electrodes.

Whilst the reference ranges are ill defined, because they are derived from only a few subjects in a narrow age range, they compare well with those of previous authors [6,11,13, 16,17].

ACKNOWLEDGEMENT

The authors wish to acknowledge the financial support of the Kidney Unit Fund, Glasgow Royal Infirmary, towards the cost of the instrument.

REFERENCES

[1] McLean, F. C. and Hastings, A. B., *Journal of Biological Chemistry*, (1935), **108**, 285.
[2] Paupa, J., These Medecine, Paris 1955.
[3] Izutsu, K. T. and Felton, S. P., *Clinical Chemistry*, 1972, **18**, 77.
[4] Izutsu, K. T., Felton, S. P., Siegel, I. A., Nicholls, J. I., Crawford, J., McGough, J. and Yoda, W. T., *Analytical Biochemistry*, 1974, **58**, 479.
[5] Robertson, W. G. and Peacock, M., *Clinica Chimica Acta*, 1968, **20**, 315.
[6] Moore, E. W., *Journal of Clinical Investigation*, 1970, **49**, 318.
[7] Pedersen, K. O., *Scandinavian Journal of Clinical Laboratory Investigation*, 1975, **35**, Suppl 143, 57.
[8] Ladenson, J. H. and Bowers, G. N., *Clinical Chemistry*, 1973, **19**, 565.
[9] Ryden, S. E., Kirkish, L. S. and McCann, D. S., *American Journal of Clinical Pathology*, 1976, **66**, 634.
[10] Fuchs, C., Dorn, D., McIntosh, C. and Scheler, F., *Clinica Chimica Acta*, 1976, **67**, 99.
[11] Madsen, S. and Ølgaard, K., *Clinical Chemistry*, 1977, **23**, 690.
[12] Fogh-Andersen, N., Christiansen, T. F., Komarmy, L. and Siggaard-Anderson, O., *Clinical Chemistry*, 1978, **24**, 1545.
[13] Ferreira, P. and Bold, A. M., *Journal of Automatic Chemistry*, 1979, **1**, 94.
[14] IUPAC Information Bulletin, 1978, No. 1, 29th General Assembly Council Meeting, Warsaw.
[15] Robertson, W.G., *Annals of Clinical Biochemistry*, 1976, **13**, 540.
[16] Husdan, H., Leung, M., Orespoulos, D. and Rapoport, A., *Clinical Chemistry*, 1977, **23**, 1775.
[17] Conceicao, S. C., Ward, M. K., Alvarez-Wde, F., Aljamma, P., Smith, P. and Kerr, D. N. S., *Clinica Chimica Acta*, 1978, **86**, 143.

### Forthcoming articles in the Journal of Automatic Chemistry

The following papers are being considered for publication in the Journal of Automatic Chemistry:–

An evaluation of the Gemsaec centrifugal analyser

Interfacing a titrator to a microcomputer for incremental or continuous modes of operation

Direct flow automated serum iron determination

Evaluation of the LKB Clinicon Chemcode system

An evaluation of the Beckman Astra 8 analyser

Relative costing of analytical systems

Particle counting immunoassay (PACIA) – its application to the determination of human placental lactogen

The flexible dialyser membrane – a source of error in plasma creatinine determination

An improved automated colorimetric analysis of fructose in fermentation media

An automated method for the determination of bromide in water

A technical evaluation of the instrumentation laboratory Multistat I analyser

